# Supplementary material for: Asynchronous magnetic resonance elastography: Shear wave speed reconstruction using noise correlation of incoherent waves
Source: Magn Reson Med. 2022 Oct 27;89(3):990–1001. doi: 10.1002/mrm.29502 (PMC9792433; doi:10.1002/mrm.29502)
Supplement: Supplementary file 1 — DATA S1 MATLAB code used to generate the numerical simulations of Figure 1 [file MRM-89-990-s002.zip › k-Wave/helpfiles/example_sd_directivity_modelling_2D.html]

Modelling Sensor Directivity In 2D Example (k-Wave)


# Modelling Sensor Directivity In 2D Example

This example demonstrates how the sensitivity of a large single element detector varies with the angular position of a point-like source. It builds on Monopole Point Source In A Homogeneous Propagation Medium and Focussed Detector In 2D examples.

- Open the file in the MATLAB Editor
- Run the file in MATLAB

## Contents

- Defining a large element detector
- Defining a collection of point sources
- Running simulations for each point source

## Defining a large element detector

The sensor is defined as a binary sensor mask in the shape of a line.

```
% define a large area detector
sz = 20;        % [grid points]
sensor.mask = zeros(Nx, Ny);
sensor.mask(Nx/2 + 1, (Ny/2 - sz/2 + 1):(Ny/2 + sz/2 + 1)) = 1;
```

## Defining a collection of point sources

Equi-spaced point sources are then defined at a fixed distance from the centre of the detector face. To do this, the Cartesian coordinates of the source points are calculated using `makeCartCircle`. A binary source mask corresponding to these Cartesian points is then calculated using `cart2grid`. The indices of the matrix elements for which the binary mask is equal to 1 (the source points) are found using `find`.

```
% define equally spaced point sources lying on a circle centred at the
% centre of the detector face
radius = 30;    % [grid points]
points = 11;
circle = makeCartCircle(radius * dx, points, [0, 0], pi);

% find the binary sensor mask most closely corresponding to the Cartesian
% coordinates from makeCartCircle
circle = cart2grid(kgrid, circle);

% find the indices of the sources in the binary source mask
source_positions = find(circle == 1);
```

A time varying pressure source is defined to drive the point sources.

```
% define a time varying sinusoidal source
source_freq = 0.25e6;   % [Hz]
source_mag = 1;         % [Pa]
source.p = source_mag * sin(2 * pi * source_freq * kgrid.t_array);

% filter the source to remove high frequencies not supported by the grid
source.p = filterTimeSeries(kgrid, medium, source.p);
```

## Running simulations for each point source

For each point source, a new binary source mask is defined, and the simulation is run. When each simulation has finished, the returned sensor data is summed together to mimic a single large detector.

```
% run a simulation for each of these sources to see the effect that the
% angle from the detector has on the measured signal
for source_loop = 1:points
    
    % select a point source
    source.p_mask = zeros(Nx, Ny);
    source.p_mask(source_positions(source_loop)) = 1;

    % create a display mask to display the transducer
    display_mask = source.p_mask + sensor.mask;

    % run the simulation
    input_args = {'DisplayMask', display_mask, 'PlotScale', [-0.5, 0.5]};
    sensor_data = kspaceFirstOrder2D(kgrid, medium, source, sensor, input_args{:});

    % average the data recorded for each grid point to simulate the
    % measured signal from a large aperture, single element, detector
    single_element_data(:, source_loop) = sum(sensor_data, 1);

end
```

The recorded time series corresponding to the different source positions are plotted below. The maximum of each time series are also plotted as a function of angle, where the angle is defined between the detector plane and a line joining the point source and the centre of the detector face. The directionality introduced by the large size of the detector is clearly seen.
